# COVID-19: reading of the Italian response to the epidemic from a historical–literary point of view

**DOI:** 10.1136/postgradmedj-2020-138138

**Published:** 2020-07-13

**Authors:** Pasquale Gallina, Marco Ricci, Paolo Lopez

**Affiliations:** University of Florence, Firenze, Italy; Court of Law, Florence, Italy; Court of Law, Cosenza, Italy

It is interesting to consider the current COVID-19 epidemic that has struck Italy, starting from the region of Lombardy, in light of the plague epidemic that exploded in Milan in 1629–1630. In certain ways, history does repeat itself.

In Alessandro Manzoni's book, *The Betrothed* (first published in 1827), perhaps the best-known historical novel to come out of Italy and required reading in secondary schools throughout the country, the author gives a vivid account of the 17th-century event. Chapters XXXII and XXXIII, in particular, describe the failure of public health policies of the times to contain the epidemic. Aided by the historiography, Manzoni reconstructed the series of devastating errors on the part of local authorities, describing the poor timing of public health interventions and, especially, the delayed and faltering establishment of a *cordon sanitaire* (our present-day ‘red zones’). Hesitancy in managing the crisis on the basis of economic considerations led to disorientation among the population, and the population reacted by putting pressure on the authorities, who in turn made a series of mistakes. One of the culminating factors was the ill-fated decision, taken under popular pressure, to organise a procession invoking saints to protect the afflicted city. Assembly of great numbers of the faithful inevitably favoured contagion, triggering an out-of-control situation. With reference to the shifting of influence between a bewildered populace and weakened governance, Manzoni wrote, ‘good sense still existed; but it was kept concealed, for fear of the popular sense’.

The Italian COVID-19 epidemic seems to offer, in a modern key, a situation similar to the sequence of events described in *The Betrothed*. Like the Milanese in Manzoni’s novel, the population seems dazed, with defects in communication—also from institutional sources—adding to the problem. For weeks prior to the outbreak in Lombardy, the population was told that COVID-19 was, all considered, little more than a simple influenza and that it almost exclusively affected the elderly and/or individuals presenting serious comorbidities.^1^ Authorities reassured the population that the measures being adopted to prevent and then limit the epidemic were the most stringent in Europe.^2^ In the coming days, citizens became aware of the much greater magnitude of the outbreak and found themselves psychologically and materially unprepared, in a stupor in the face of the collapse of the healthcare system in Lombardy, among the top in the country.^3^After diagnosis (on 21 February 2020) of the first COVID-19 patient in Codogno, near Milan, social distancing measures were put into effect in several northern Italian areas.^4^ Many individuals outside the red zones, unaware of the severity of the epidemic, travelled freely between one region and another, spreading the infection. Meanwhile, comments circulated among the citizenry and business about the inconvenience of restrictive provisions that limited personal freedoms and production.^5^ Also, these pressures may have played a role in the fact that restrictions were hesitatingly progressive and asynchronistic on a national level. It is true that religious processions were not organised as in 17th-century Milan; however, suspension of sporting events, such as football matches, which bring together thousands of people in our country, was ordered only on 9 March^6^ due to concerns about the impact on the football industry, in economic terms, as well as on the general mood of the population. Compounding the situation, workers circulated on crowded public transportation since there were no broad guidelines regarding workplaces. It was only with the drastic measures set out by the government’s decree^7^ on 21 March that all unjustified movements were forbidden and that all non-essential work was suspended. Paradoxically, the ill-advised release of the contents of the decree^7^ on the day prior to its issue, a repeated mistake,^8^ led to an uncontrolled, biblical exodus from northern industrial areas toward the south, the land of origin of many industrial workers who impulsively fled the regions of contamination towards those apparently spared from COVID-19.^9^

Intuitively, adoption of drastic policy decisions that balance public health and economic and social needs is difficult. The necessity to pursue and maintain consensus, inherent in any democratic political system, can become a danger for current democracies due to constant media coverage.^10^ The specific Italian approach to managing the crisis and the effectiveness of policies to combat the epidemic should be the subject of future studies. However, a timely and honest debate among the international scientific community about the appropriateness or not of the public health decisions carried out by our country in the face of COVID-19 is needed. Non-tryrannical leaders need solid consensus for difficult undertakings in order to get results. In light of the seriousness of the moment and the landmark reach of the events, we cannot wait for historic judgement, as in the case of Milan’s 17th-century plague, but need to also consider the leadership’s choices in the face of public opinion.
